# Editorial: Influenza Virus Vaccines and Immunotherapies

**DOI:** 10.3389/fimmu.2015.00599

**Published:** 2015-11-24

**Authors:** Arun Kumar, Shakti Singh

**Affiliations:** ^1^GlaxoSmithKline Vaccines, Research Center, Siena, Italy; ^2^Department of Surgery, University of Alberta, Edmonton, AB, Canada

**Keywords:** influenza, human, influenza A virus, vaccines, influenza vaccines, immunotherapy

Influenza virus infections are responsible for billions of dollars of economic burden annually worldwide. Regardless of advances in our understanding of the immune evasion mechanism, the disease remains one of the foremost threats for human being (1). Currently available vaccines and therapeutic agents are not very potent against the deadly flu infections because of the frequent mutations in influenza virus subtypes. The mismatch between the circulating strains and vaccine strains critically affects the success rate of the conventional vaccines and requires continuous monitoring of circulating influenza strains. These collective limitations of existing flu vaccines urgently call for the development of novel vaccines with a wide range of cross-protective immunity (2). The discovery and development of new and improved vaccines have been greatly facilitated by the application of new technologies. The articles included in this research topic explain the key methods of improvement in existing vaccines, therapeutics, and mechanism of protection.

The opening articles of the present topic discuss about the novel methods of improving existing influenza vaccines and enhancing immune responses. Various strategies including use of adjuvants, heterologous prime/boost and unique antigen design have shown to induce protective influenza-specific neutralizing antibodies. Lofano et al. reviewed recent advancements in the flu vaccine development and highlight the role of B-cells in controlling influenza virus infections (3). Soema et al. discussed the recent developments and design of T-cell based vaccines based on novel peptide and protein-based vaccine formulations (4). Adjuvants play a critical role in the induction of rapid, effective, and durable immune responses when administered with vaccine antigens. Recent progress in the adjuvant formulations allows existing vaccines to reduce the number of booster doses, increase dose-sparing ability, induce potent T and B-cell responses and enhance breadth of the immune responses against heterotypic antigens (5). Vogel and Brown reported that only single dose of CpG adjuvanted influenza vaccine can induce robust memory T-cell responses and confer protection against heterosubtypic challenge (6). Similarly, in the presence of oil-in-water emulsion adjuvant system (AS03), even a very small dose of influenza vaccine can induce significantly strong immune responses (7). Alternative strategies of immune protection such as use of immuno-modulatory agents like TLR agonist can activate protective non-specific antiviral immune responses. Mifsud et al. demonstrated that mice pre-treated with TLR-2 agonist PEG-Pam2Cys were able to mount specific B- and T-cell responses and also protect mice against heterologous virus challenge (8). These types of alternative strategies can provide immediate immune protection in the absence of effective vaccine without compromising the antiviral specific immunity.

The next generation universal influenza vaccine targets the most conserved structure of the virus and hence confers heterotypic protection, for example, new vaccine strategies target stalk of the HA instead of the globular head. Mallajosyula et al. designed a potent immunogen comprised of HA-stem-fragment from H3N2 strain (A/Hong Kong/1/68) and trimerization motifs: coiled-coil isoleucine zipper and globular β-rich. Immunization of mice with the immunogen induced cross-reactive antibodies and provided only partial protection against homologous virus challenge (9). Li et al. engineered a dual vaccine by incorporating botulinum neurotoxin A (BoNT/A) receptor-binding subdomain in universal influenza vector based on PR8 strain of influenza virus. This vaccine induced protective immunity against both BoNT/A and Influenza virus. Potent immune responses can also be generated by exploiting receptors on antigen presenting cells (APCs) (10). Grodeland et al. demonstrated that a novel DNA vaccine candidate specifically induced Th2 and IgG1 antibody responses or Th1 and IgG2a responses by targeting hemagglutinin to MHC class II molecules or chemokine receptors (XCR1 or CCR1/3/5), respectively (11).

The historical 1918 influenza pandemic caused very high mortality in adult population and the immunological parameters in this population still remain speculative. In light of current understanding of influenza immuno-pathogenesis, McAuley et al. claimed that, the high mortality rate in the adult population is due to the dysfunctional or excessive cross-reactive memory T-cells, induced by previous influenza infections, which render these individual susceptible to the 1918 pandemic influenza (12). Peng et al. provided the evidence that pre-existing seasonal influenza virus HA-specific cross-reactive T-cells can be boosted by a heterologous vaccine (13). Pregnant women are at paramount priority for influenza vaccination due to influenza virus related complications during the pregnancy. Modulation of maternal immunity during the pregnancy can influence the influenza vaccine specific immune responses. Kay and Blish summarize the immunogenicity and efficacy of the influenza vaccine and discuss impact on T and B-cell responses during the pregnancy (14).

Apart from prophylactic vaccine, therapeutic approaches also required for those severely infected with the influenza virus. The use of antibodies seems to be an attractive immunotherapeutic approach for the treatment of various infectious diseases. Sasisekharan et al. reviewed different antibody based strategies to prevent and treat the influenza infections (15). The cells of innate immune system are triggered by signaling pathways during influenza virus infection that causes extensive damage to lung tissues and in airway lining, resulting in severe immunopathology. Ramos and Sesma suggest that these damages can be minimized by selectively modulating the innate signaling pathways using immuno-modulatory drugs while maintaining the ability of the host cells to mount an antiviral response to control virus replication (16). Further, Graham et al. described the role of mast cells in immunopathology during influenza A virus infection and suggested them as a potential drug target in viral infections (17).

The new strategies for vaccine design also require more sensitive and efficient methods for the evaluation of the vaccine potency. Carnell et al. reviewed comprehensive methods for influenza virus neutralization assays, based on the pseudotype viral particles, which utilize chimeric viruses bearing influenza glycoproteins, and depict how such assays can replace the traditional HA neutralization assays for the evaluation of new age influenza vaccines (18). To detect the incidence of Influenza virus with high efficiency and accuracy, Rajput et al. generated HA specific high affinity recombinant single chain variable fragment (scFv) antibodies showing high sensitivity (83.9%) and specificity (100%) for three different strains of influenza virus (19).

The compilation of research articles included in this research topic should help reader to have an overview of different strategies for improving existing influenza vaccines and immunotherapies. This research topic also highlights the progress made in understanding of the immune protection and pathogenic mechanisms of influenza virus.

## Conflict of Interest Statement

The authors declare that the research was conducted in the absence of any commercial or financial relationships that could be construed as a potential conflict of interest.
